# Impact of Hypertension on the Dose-Response Association Between Physical Activity and Stroke: A Cohort Study

**DOI:** 10.1161/STROKEAHA.123.045870

**Published:** 2024-08-08

**Authors:** Hannah L. McLellan, Ellen A. Dawson, Thijs M.H. Eijsvogels, Dick H.J. Thijssen, Esmée A. Bakker

**Affiliations:** Liverpool Centre for Cardiovascular Science, Research Institute for Sport and Exercise Science, Liverpool John Moore’s University, United Kingdom (H.L.M.L., E.A.D., D.H.J.T.).; Department of Medical BioSciences, Radboud University Medical Center, Nijmegen, the Netherlands (T.M.H.E., D.H.J.T., E.A.B.).; Department of Physical Education and Sports, Sport and Health University Research Institute (iMUDS), University of Granada, Spain (E.A.B.).

**Keywords:** health, hypertension, inactivity, regular physical activity, stroke risk

## Abstract

**BACKGROUND:**

Regular physical activity is associated with a reduced stroke risk. However, this relationship might be attenuated in the presence of hypertension and antihypertensive medication use. We examined the dose-response relationship between physical activity and stroke in normotensive and hypertensive individuals.

**METHODS:**

A Dutch population-based cohort including 139 930 individuals (41% men; mean age, 44±13) was performed (median follow-up, 6.75 years). Participants were stratified at baseline as hypertensive (44%) or normotensive (56%) and categorized into quartiles of the lowest (Q1) to the highest (Q4) moderate-to-vigorous, self-reported physical activity. The primary outcome was incident stroke (fatal and nonfatal). Cox regression estimated hazard ratios and 95% CIs. The main analyses were stratified on baseline blood pressure and adjusted for confounders. Hypertensives were stratified into medicated (21%) or non-medicated (79%).

**RESULTS:**

Compared with Q1, adjusted hazard ratios were 0.87 (0.69–1.10; *P*=0.23), 0.75 (0.59–0.95; *P*=0.02), and 0.94 (0.74–1.20; *P*=0.64) for Q2 to Q4, respectively in the total population. Hazard ratios for normotensives were 0.79 (0.50–1.25; *P*=0.32), 0.75 (0.48–1.18; *P*=0.22), 0.97 (0.62–1.51; *P*=0.90) for Q2 to Q4, respectively. In hypertensives, hazard ratios were 0.89 (0.68–1.17; *P*=0.41), 0.74 (0.56–0.98; *P*=0.03), 0.92 (0.69–1.23; *P*=0.56) for Q2 to Q4, respectively. There was no significant interaction between hypertension status for the relation between physical activity and stroke risk. The stratified analysis revealed a smaller benefit of moderate-to-vigorous physical activity in medicated hypertensives compared with nonmedicated hypertensives, but no significant interaction effect was found.

**CONCLUSIONS:**

Regular moderate-to-vigorous physical activity is beneficial for stroke risk reduction (Q3 compared with Q1), which is not affected by hypertension. Antihypertensive medication may be associated with a smaller benefit of moderate-to-vigorous physical activity on the risk of stroke, but further research is warranted.

Stroke remains a significant health problem, with a predicted increased incidence of 34% between 2015 and 2035.^1^ This highlights the need for preventive strategies to lower the risk for stroke, and thereby reducing the health and socioeconomic effects pertaining to stroke.

Studies have found potential health benefits of physical activity (PA) in the prevention of stroke in the general population.^2,3^ PA is defined as any bodily movement that is performed by the muscles that require energy and the 2020 World Health Organization PA Guidelines recommend adults perform at least 150 minutes/wk of moderate-intensity PA, or 75 minutes/wk of vigorous-intensity PA, or an equivalent combination of the 2.^4^ For example, ≥30 minutes of moderate-to-vigorous PA during 1× to 2× per week resulted in a 16% risk reduction for the risk of stroke compared with inactive individuals.^5^ Similarly, 3× to 4× times per week and ≥5× per week of MVPA sessions were also associated with a 21% and 22% lower risk of stroke compared with inactivity, respectively.^5^ In addition to the risk of stroke, MVPA is also associated with a lower risk of developing hypertension, an important modifiable risk factor for stroke.^6^ Some studies have suggested that the positive responses to PA may become stronger with the prevalence of risk factors, such as hypertension.^7–11^ Supporting this, a recent study in 142 493 adults found that individuals with cardiovascular risk factors may have larger benefits of MVPA,^12^ as the dose-response association between MVPA and major cardiovascular events and mortality became stronger and contained larger (relative) risk reductions in individuals with risk factors compared with individuals without risk factors or with established CVD. The dose-response relation between PA and the risk for stroke, therefore, may be affected by the presence of hypertension, one of the key risk factors. Furthermore, antihypertensive pharmacological treatments may also interfere with the benefits of exercise training on the risk of clinical events.

Therefore, the aim of our study was to examine the dose-response association between MVPA and stroke and to determine whether this association differed between those with a priori hypertension and normotension. Second, we aimed to evaluate whether the use of antihypertensive medication alters this dose-response association. In line with recent work, we expect that those with hypertension will demonstrate a stronger inverse association between MVPA and the risk of stroke. In addition, we hypothesize that antihypertensive medication may attenuate the benefits of MVPA on stroke, as recent exercise training studies evaluating the impact of drugs on exercise-mediated cardiovascular adaptations were blunted angiotensin-converting enzyme inhibitors while angiotensin-converting.^13^

## METHODS

### Study Population

This study used data from the Lifeline Cohort Study. Lifelines is a multidisciplinary prospective population-based cohort study examining in a unique 3-generation design the health and health-related behaviors of 167 729 persons living in the North of the Netherlands. It employs a broad range of investigative procedures in assessing the biomedical, sociodemographic, behavioral, physical, and psychological factors, which contribute to the health and disease of the general population, with a special focus on multi-morbidity and complex genetics.^13,14^ All individuals who lived in the northern Netherlands were eligible to take part, other than those with (1) severe psychiatric or physical illness (eg, individuals with cancer and associated reduced life expectancy), (2) life expectancy <5 years; and (3) lack of fluency in Dutch. Participants who were ≥18 years old were included (N=152 737). Participants were excluded (N=12 807) due to (1) missing data for PA, blood pressure, and CVD health status; (2) limited ability to be physically active; (3) inability to merge individual data with registry data; and (4) the presence of cardiovascular disease (Figure S1). More detailed information about the response rate for each time point can be found elsewhere.^15^ The Strengthening the Reporting of Observational Studies in Epidemiology guideline was used to report our findings (Table S1). All participants provided written informed consent. The Lifelines Cohort study was conducted according to the principles of the Declaration of Helsinki and approved by the University Medical Center Groningen Medical Ethical Committee.

### Data Availability

The data of the study cannot be shared publicly due to contractual restriction of the Data Use Agreement of the Lifelines Study and Statistics Netherlands. Researchers wanting to obtain or use data can contact the Lifelines study team (https://www.lifelines-biobank.com/) and Statistics Netherlands (https://www.cbs.nl/en-gb/our-services/customised-services-microdata/microdata-conducting-your-own-research). Data cleaning, processing, and analyses scripts are available upon request to the corresponding author.

### Measurements

#### Physical Examination and Questionnaire

Participants visited one of the Lifeline research sites for a physical examination and completed a baseline questionnaire between 2006 and 2013. Baseline data were collected for 167 729 participants, aged from 18 to 93 years. Every 1.5 years, a follow-up questionnaire was administered to assess the occurrence of a stroke.

The physical examination included an anthropometric assessment, blood pressure (BP) measurements, and blood draw. Anthropometrics included height (stadiometer) and weight (standard weighing scale), which were used to calculate body mass index (kg/m^2^). Ten BP measurements were taken during a 10-minute period using an automated sphygmomanometer (Dynamap, PRO 100, or PRO 100V2) placed around the upper right arm (or the left arm if contraindications were present) with participants in a seated position, the 2 last successive BPs were averaged. Blood samples were taken after >8-hour fasting for measurements of LDL (low-density lipoprotein), glucose, and serum creatinine. Renal function (estimated glomerular filtration rate) was estimated.^16^

Baseline questionnaires included questions on demographics, health status, and lifestyle. Demographics included age, sex, postal code, income, and education level. Income was estimated using the postal codes and data of Statistics Netherlands^17^ when not reported. Education level was categorized as low, moderate, and high. Medical history included self-reported information on medication use, presence of CVD, comorbidities, and other illnesses including cancer, arthritis, multiple sclerosis, and Parkinson disease. Lifestyle factors included smoking status, alcohol consumption, and hours of sleep per night. Smoking status was categorized as currently, previously, and never. High alcohol consumption was defined as >14 drinks/wk or >4 drinks/d for men and >7 drink/wk or >3 drink/d for women.^18^

#### Habitual PA Volume

Baseline PA was assessed using the Short Questionnaire to Assess Health-enhancing PA. Short Questionnaire to Assess Health-enhancing PA divides habitual PA into transportation, occupation, household, and leisure domains and asks for the duration and intensity of an individual’s typical weekly activities over the past 3 months.^19^ Weekly physical activities were converted to the average amount of metabolic equivalent of task (MET) minutes per week based on the Compendium of Physical Activities.^20^ MET minutes were calculated by multiplying the MET values of each activity by the duration (ie, minutes per week). Only activities with ≥3 MET value were included since these activities relate to moderate-to-vigorous activities as specified in the World Health Organization PA guidelines.^21^ Individuals were categorized into 4 quartiles of least (Q1) to most (Q4) physically active based on self-reported MVPA volumes. Q1 included the least active individuals who spent <1830 MET min/wk, Q2 included individuals with 1830 to 3617 MET min/wk, Q3 included individuals with 3618 to 7175 MET min/wk, and Q4 included individuals with >7175 MET min/wk.

#### Clinical Outcomes

The primary end point was the occurrence of a stroke. Hospital registry data of statistics Netherlands were used to determine the primary outcome. When hospital registry data were not available, self-reported stroke during follow-up were used instead. Self-reported stroke was measured after a median follow-up of 1.1, 2.1, and 3.8 years. The date on which the questionnaire was completed was used for the event date of self-reported stroke. Participants were followed until the first stroke event. Participants who did not reach the end point were censored at the end of the last assessment or at the date they died, whichever occurred first.

### Hypertension

#### Hypertension

Participants were divided as hypertensive or normotensive based on the BP evaluation performed at baseline, while using the recent classifications of hypertension following the updated National Institute for Health Care Excellence guidelines (ie, ≥130/80 mm Hg). In addition, those who reported being diagnosed by a physician with hypertension and used BP-lowering medication were included in the hypertensive group. Those who did not report being diagnosed with hypertension and had a baseline BP of ≤129/79 mm Hg were categorized into the normotensive group.

#### Medicated

For our secondary analysis, we have divided the hypertensive group into a medicated and nonmedicated group. Individuals were categorized into the medicated group if they were prescribed ACE (angiotensin-converting enzyme) inhibitors, angiotensin blockers, beta-blockers, diuretics, or calcium antagonists.

### Statistical Analyses

Baseline characteristics were described for the normo- and hypertensive group. Normally distributed data were presented with mean (±SD) and non-normally distributed data were presented with the median (interquartile range, Q_25_–Q_75_).

Stratified Kaplan-Meier curves and log-rank tests were performed to assess differences in the outcome between the quartiles of PA. A Multivariable Cox regression model was conducted to estimate the association between PA and stroke using hazard ratios (HRs) with 95% CIs. First, an unadjusted model was performed (model 1). Model 2 was adjusted for age (years), sex (male/female), body mass index (kg/m^2^), income (per 1000 euros), an education level (low/moderate/high), smoking (pack years), kidney function (glomerular filtration rate, mL/min per 1.73m^2^), glucose (mmol/L), and LDL (low-density lipoprotein, mmol/L). Model 3 included the variables of model 2 and was additionally adjusted for systolic BP (mm Hg), diastolic BP (mm Hg), use of acetylsalicyclic acid, antiplatelets, and antihypertensive medication. The analyses were performed for the total population and were stratified for hypertensive and normotensive individuals. We also tested interaction in terms of BP (normotensive versus hypertensive) and the 4 quartiles of MVPA. In addition, to examine whether medication use altered the dose-response association of MVPA in hypertensive individuals, we performed stratified analyses and tested the interaction term.

Since the total number of participants with missing data (from all variables) was relatively small (<14%), the complete case was used for the analyses. All statistical analyses were performed in R version 3.5.2. *P*<0.05 were considered statistically significant.

## RESULTS

### Study Population

**Figure 1.**
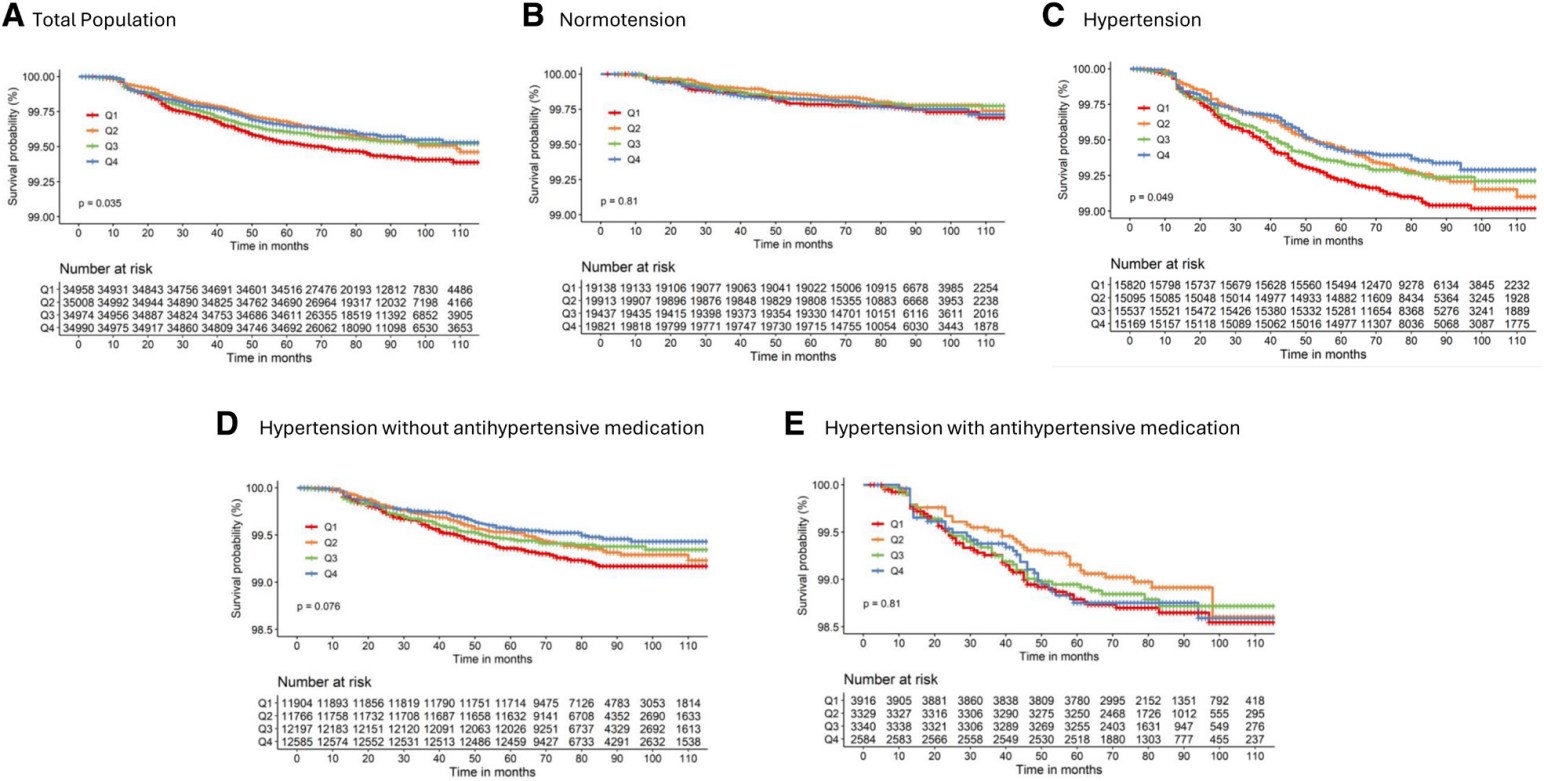
**Unadjusted Kaplan-Meier estimates of stroke occurrence for quartiles (Q) of moderate-to-vigorous physical activity during follow-up.** Kaplan-Meier plots were stratified for total population (**A**), individuals with normotension (**B**), and individuals with hypertension (**C**), individuals with hypertension who are not taking antihypertensive medication (**D**) and individuals with hypertension who are taking antihypertensive medication (**E**). *P* values of the log-rank tests examining differences between Kaplan-Meier curves of the different quartiles of physical activity are provided in each graph.

In total, 139 930 participants were included in our analysis. Forty-one percent of the population was male with an overall mean age of 41 (13) years (Table 1). The median follow-up was 6.75 years (Q_25_, 5.83; Q_75_, 7.92) with a total number of 640 strokes (0.46%; Figure 1). Individuals with hypertension were more often male, older, had a lower education level and higher body mass index, used more often medication, and had more comorbidities. Within the hypertensive group, medicated individuals were more often female, older, had a lower education level, and had more comorbidities compared with nonmedicated (Table S2).

**Table 1.**
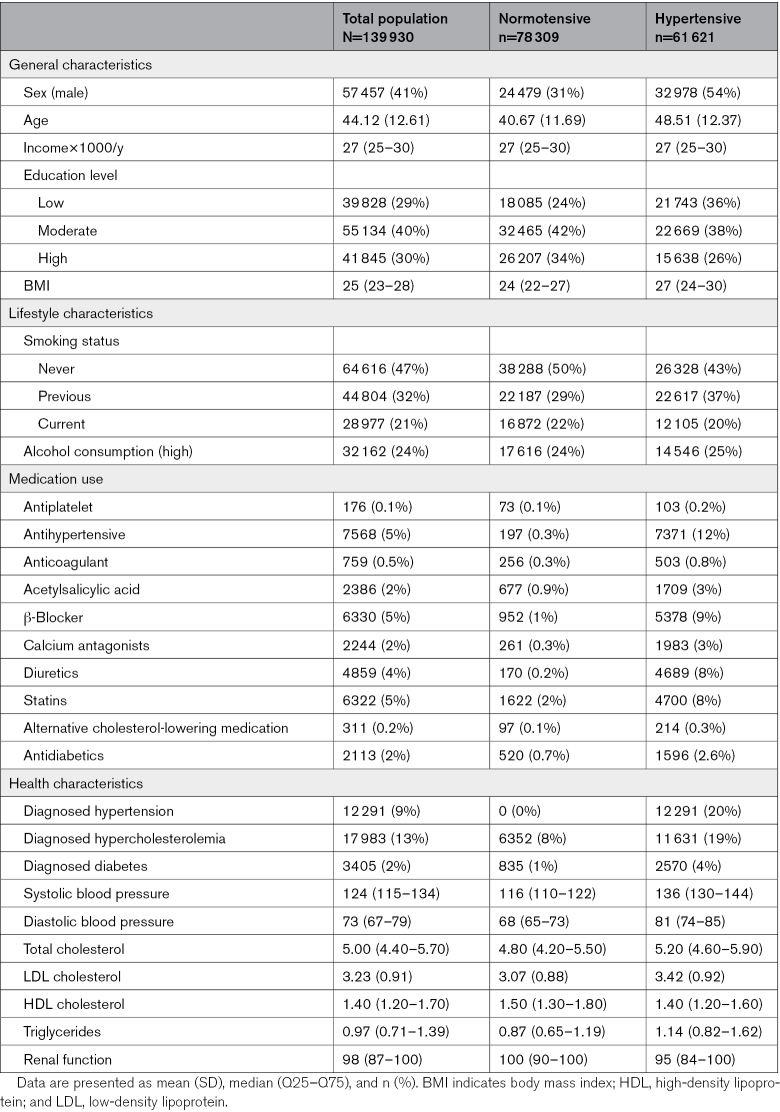
Baseline Characteristics for the Total Population (N=139 949) and Individuals With Normotension (n=78 309) and Hypertension (n=61 621)

### PA and the Risk of Stroke

**Figure 2.**
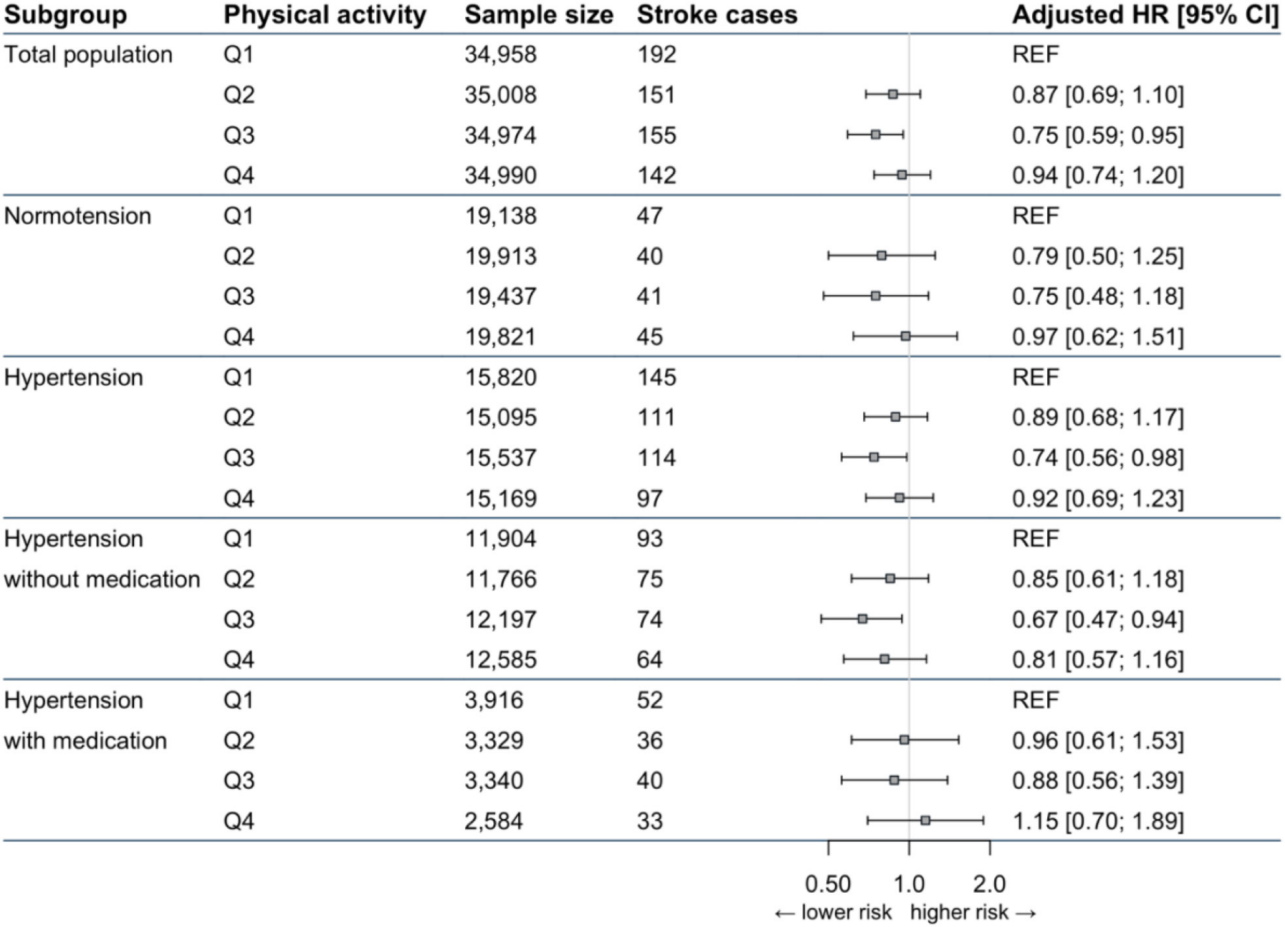
**Forest plot of the quartiles (Q) of moderate-to-vigorous physical activity associated with stroke risk for the total population and stratified for blood pressure and medication use.** Hazard ratios (HRs) were adjusted for age, sex, body mass index, income, education level, alcohol, smoking (pack years), kidney function, body mass index, serum glucose, systolic blood pressure, diastolic blood pressure, low-density lipoprotein cholesterol, use of acetylsalicylic acid, antiplatelets, and antihypertensive medication (model 3).

We found a trend for a negative, linear relation between MVPA and stroke risk for the total population and in the hypertensive subgroup. After adjusting for confounding factors, no significant association was observed (models 2 and 3; Table 2). After adjustments for confounders (model 3) and using MVPA categories, a significantly lower HR was found in Q3 compared with the least active individuals (Q1) within the total population and in individuals with hypertension (Table 2; Figure 2). No significant association was found in Q2 and Q4 compared with Q1. Although we found no significant association between MVPA and stroke risk in normotensive individuals, estimated HRs were largely comparable to the hypertensive participants. In addition, there was no significant interaction between each of the MVPA quartiles for stroke risk and normotension versus hypertension (*P*>0.05).

**Table 2.**
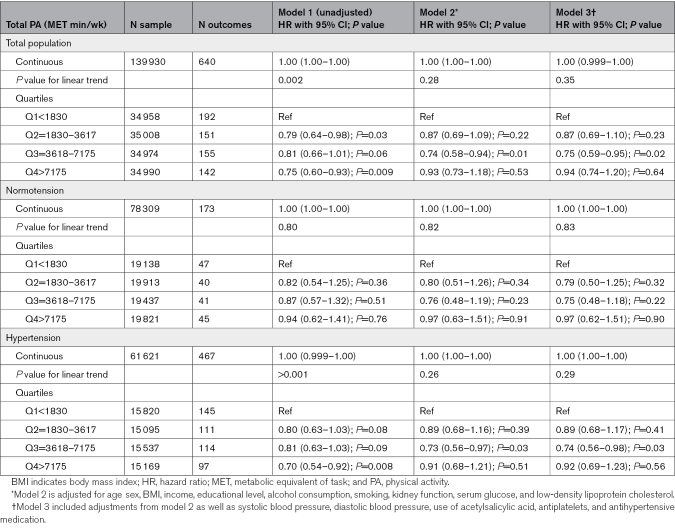
HRs With 95% CIs for the Association Between Moderate-to-Vigorous Physical Activity and Stroke

### PA and Stroke Risk in Hypertensives: Impact of Medication Use

Within hypertensive individuals, after adjustment for confounders, there was a significant reduction for Q3 compared with Q1 for MVPA and stroke risk in those without medication (Table 2; Figure 2). In medicated hypertensive individuals, no significant association was found between PA and stroke risk, with estimated HRs being slightly higher compared with the nonmedicated hypertensive population (Table 2; Figure 2). However, the *P* value for interaction between PA and medication use was nonsignificant (*P*>0.05).

## DISCUSSION

Previous research has demonstrated the importance of PA on stroke risk reduction; however, emerging research has suggested that the dose-dependent association of regular PA may be altered in the presence of cardiovascular risk factors and medication use. This study presents the following findings. First, we reinforce the benefits of regular PA, as engagement in MVPA is associated with a lower risk for stroke, which remained significant upon adjustment for potential confounders. Second, the shape of the dose-response association does not significantly differ between hypertensive (n=61 621) and normotensive (n=78 309) individuals. However, the association remained only significant in the hypertensives after stratification. Third, the benefits of regular MVPA seem to be only stronger in nonmedicated individuals with hypertension, but further studies are necessary to confirm this preliminary result. Our findings highlight that a physically active lifestyle is beneficial for reducing stroke risk.

Our study reinforces findings from previous studies highlighting that more PA is associated with a reduction in the risk for stroke in the general population. For example, a study in the United States, with a median follow-up of 18.8 years and 648 ischemic stroke events, showed a significant reduction in ischemic stroke (and other subtypes) with increasing PA levels among middle-aged men and women. Another prospective cohort study in South Koreans with 13 years of follow-up showed that exercising between 3 and 4 or 5 to 6 times per week showed the lowest stroke risk compared with physically inactive individuals.^22^ In contrast to others, our study further examined whether the association between MVPA and stroke depends on BP levels. We found that the shape of the dose-response association between MVPA and stroke risk was not significantly different between individuals with normotension or hypertension. Nevertheless, the association was not significant in the normotensive group. A possible explanation for this observation is due to the low event rate present in the normotensive group (ie, >60% less events in the normotensives versus the hypertensives). In addition, compared with other studies, our follow-up time was shorter (ie, median 6.8 years) and we included a relatively young population, which suggests that a longer follow-up in the normotensives who have a lower a priori risk for stroke may be required. Furthermore, HRs in Q4 in the present study are higher than in Q3; however, this may be explained by possible measurement error in MVPA classification, Specifically, Q4 may contain individuals who exaggerated their PA levels but due to the nature of questionnaires and the absence of an objective PA measurement we can only speculate. Importantly, our findings show that the presence of hypertension does not attenuate the dose-response relationship between MVPA and stroke. This finding supports previous studies examining the effect of PA and cardiovascular disease and mortality.^12,23^ Our study provides an important public health message; that benefits from a physically active lifestyle in relation to stroke remain equally present in those who have already developed hypertension.

For our secondary aim, we compared the effects of PA on stroke risk between medicated and unmedicated hypertensive individuals. While reinforcing our initial results, in that PA is associated with lower risks for stroke in unmedicated hypertensive individuals, such effects were not observed in medicated individuals with hypertension. One potential explanation of the latter observation is that the sample size and event rate of the medicated hypertensives was relatively low. Although the medicated hypertensive individuals might be underpowered, the analysis showed higher HRs in this subgroup, which might suggest an attenuated and smaller effect of MVPA on the risk of stroke in the medicated group of individuals with hypertension. One potential explanation could be that those within the medicated group had higher BP levels and disease severity. Previous research has suggested that those with established CVD may not benefit to the same extent as healthy individuals.^10–12^ In addition, the attenuated effect may relate to the use of medication itself. However, previous work is conflicting on whether antihypertensive medication may interfere with or potentiate the benefits of PA or exercise training.^24–27^ Apart from the antihypertensive medication, other drugs that are commonly prescribed to hypertensive individuals such as statins (21% in our medicated hypertensives) may also have an effect on the benefits of PA.^28^ Therefore, further research is warranted to understand whether antihypertensive medication affects the benefits of PA on stroke risk. Nonetheless, we think it is important to emphasize that, even in the presence of hypertension and medication use, other research has suggested that when combined, exercise and medication strengthen the BP effects of medication alone and should be recommended and implemented as indicated according to existent professional treatment algorithms.^29,30^

### Strengths and Limitations

A particular strength of this study is that it includes a large population (N=139 930) with outcome data based on national hospital registry data. One potential limitation of our study is that MVPA was self-reported, which is susceptible to overestimating true PA volumes.^31^ Nonetheless, categorizing individuals across quartiles allowed comparison between the least active individuals and those engaged in larger volumes of MVPA, and ultimately self-reported MVPA data are likely to underestimate the true effect of MVPA on health benefits.^31^ Furthermore, we did not measure light-intensity PA. Although MVPA has larger health benefits than light-intensity PA,^32^ it is important to acknowledge that it may still have a positive effect on stroke risk. However, the validity of recalling light-intensity PA using subjective tools such as questionnaires is poor due to recall bias with previous validation studies highlighting that the validity of questions is higher for higher levels of PA.^33^ In addition, this study did not collect data on inactive behaviors such as sedentary time. However, given that physical inactivity is an independent predictor of all-cause mortality and morbidity^34^ future work should include this. Another limitation relates to the relatively low event rate of stroke in the normotensive population and the relatively small sample size of medicated hypertensive individuals. Nonetheless, HRs in both populations, albeit with greater confidence intervals, provide insight into the effects of PA.

### Perspectives

Our study reinforces the benefits of regular MVPA on stroke risk reduction. However, the adjusted dose-response association was only statistically significant for Q3 compared with Q1. We found that the shape of the dose-dependent association between MVPA and risk of stroke was not different between individuals with hypertension and normotension. Furthermore, our data provides preliminary support that in individuals with hypertension, the use of antihypertensive medication may be associated with a smaller benefit of MVPA on the risk of stroke. Future studies are warranted to better understand the dose-response association of MVPA on the risk of stroke including the impact of antihypertensive medication and the potential underlying mechanisms that could drive these effects. Such information can further optimize MVPA prescription in the prevention of stroke.

## ARTICLE INFORMATION

### Acknowledgments

The authors wish to acknowledge the services of the Lifelines Cohort Study, the contributing research centers delivering data to Lifelines, and all of the study participants.

### Sources of Funding

Dr Bakker has received funding from the European Union’s Horizon 2020 research and innovation program under the Marie Skłodowska-Curie grant agreement No (101064851). The Lifelines Biobank initiative received funding from the Dutch Ministry of Health, Welfare and Sport, the Dutch Ministry of Economic Affairs, the University Medical Center Groningen (UMCG), University Groningen, and the Northern Provinces of the Netherlands. The funders had no role in study design, data collection and analysis, decision to publish, or preparation of the article.

### Disclosures

None.

### Supplemental Material

Tables S1–S2

Figure S1

## Supplementary Material


