# Supplementary material for: Impact of Hypertension on the Dose-Response Association Between Physical Activity and Stroke: A Cohort Study
Source: Stroke. 2024 Aug 8;55(9):2231–9. doi: 10.1161/STROKEAHA.123.045870 (PMC11346707; doi:10.1161/STROKEAHA.123.045870)
Supplement: Supplementary file 1 [file str-55-2231-s001.pdf]

Supplemental material

Table S1 STROBE Checklist.
Strengthening the Reporting of Observational Studies in Epidemiology.

	Item No	Recommendation	Page No
**Title and abstract**	1	(*a*) Indicate the study's design with a commonly used term in the title or the abstract<br>(*b*) Provide in the abstract an informative and balanced summary of what was done and what was found	Title and abstract paragraph 2<br>abstract
**Introduction**			
Background/rationale	2	Explain the scientific background and rationale for the investigation being reported	Introduction, paragraph 1-3
Objectives	3	State specific objectives, including any prespecified hypotheses	Introduction, paragraph 3
**Methods**			
Study design	4	Present key elements of study design early in the paper	Study population, paragraph 1
Setting	5	Describe the setting, locations, and relevant dates, including periods of recruitment, exposure, follow-up, and data collection	Study population , paragraph 1
Participants	6	(*a*) Give the eligibility criteria, and the sources and methods of selection of participants. Describe methods of follow-up<br>(*b*) For matched studies, give matching criteria and number of exposed and unexposed	Study population, paragraph 1 and Clinical outcomes, paragraph 1<br>-
Variables	7	Clearly define all outcomes, exposures, predictors, potential confounders, and effect modifiers. Give diagnostic criteria, if applicable	Methods, all paragraphs
Data sources/ measurement	8*	For each variable of interest, give sources of data and details of methods of assessment (measurement). Describe comparability of assessment methods if there is more than one group	Methods, all paragraphs
Bias	9	Describe any efforts to address potential sources of bias	Statistical analyses, all paragraphs
Study size	10	Explain how the study size was arrived at	Study population, paragraph 1
Quantitative variables	11	Explain how quantitative variables were handled in the analyses. If applicable, describe which groupings were chosen and why	Statistical analyses, paragraph 2
Statistical methods	12	(*a*) Describe all statistical methods, including those used to control for confounding<br>(*b*) Describe any methods used to examine subgroups and interactions<br>(*c*) Explain how missing data were addressed<br>(*d*) If applicable, explain how loss to follow-up was addressed<br>(*e*) Describe any sensitivity analyses	Statistical analyses, all paragraphs<br>Statistical analyses, paragraph 2 to 4<br>Statistical analyses, paragraph 3<br>Clinical outcomes, paragraph 1<br>Statistical analyses, paragraph 4
**Results**			

Participants	13*	(a) Report numbers of individuals at each stage of study—eg numbers potentially eligible, examined for eligibility, confirmed eligible, included in the study, completing follow-up, and analysed	Study population (methods), paragraph 1 and Study population (results), paragraph 1
		(b) Give reasons for non-participation at each stage	Study population (methods), paragraph 1 and Study population (results), paragraph 1 and
		(c) Consider use of a flow diagram	S1 fig
Descriptive data	14*	(a) Give characteristics of study participants (eg demographic, clinical, social) and information on exposures and potential confounders	Study population (results), paragraph 1 and table 1
		(b) Indicate number of participants with missing data for each variable of interest	-
		(c) Summarise follow-up time (eg, average and total amount)	Clinical outcomes, paragraph 1
Outcome data	15*	Report numbers of outcome events or summary measures over time	Clinical outcomes, paragraph 1
Main results	16	(*a*) Give unadjusted estimates and, if applicable, confounder-adjusted estimates and their precision (eg, 95% confidence interval). Make clear which confounders were adjusted for and why they were included	Health benefits of MVPA to Dose-response relationship of domain-specific MVPA and Tables 2, S2 t/m S12
		(*b*) Report category boundaries when continuous variables were categorized	Statistical analyses paragraph 2
		(*c*) If relevant, consider translating estimates of relative risk into absolute risk for a meaningful time period	-
Other analyses	17	Report other analyses done—eg analyses of subgroups and interactions, and sensitivity analyses	Health benefits of MVPA, paragraphs 2 and 3
**Discussion**			
Key results	18	Summarise key results with reference to study objectives	Discussion, paragraph 1
Limitations	19	Discuss limitations of the study, taking into account sources of potential bias or imprecision. Discuss both direction and magnitude of any potential bias	Strengths and limitations, paragraph 1
Interpretation	20	Give a cautious overall interpretation of results considering objectives, limitations, multiplicity of analyses, results from similar studies, and other relevant evidence	Cardiovascular health status and MVPA benefits to Leisure versus non-leisure MVPA
Generalisability	21	Discuss the generalisability (external validity) of the study results	Cardiovascular health status and MVPA benefits to Strengths and limitations
**Other information**			
Funding	22	Give the source of funding and the role of the funders for the present study and, if applicable, for the original study on which the present article is based	Title page, funding/support

*Give information separately for exposed and unexposed groups.

Table S2. Baseline table of individuals with hypertension

General characteristics	Hypertensives without medication (N=48,452)	Hypertensives with medication (N=13,169)
Sex (male)	27,584 (57%)	5,394 (41%)
Age	46.43 (11.90)	56.16 (10.95)
Income x 1000/year	27 [25.00, 30.00]	26.80 [24.90, 29.70]
Education level		
low	15797 (33%)	5,946 (47%)
moderate	18620 (39%)	4,049 (32%)
high	12943 (27%)	2,695 (21%)
BMI (median [IQR])	26.40 [24.20, 29.10]	27.90 [25.40, 31.10]
**Lifestyle characteristics**		
Smoking status		
Never	21,246 (44%)	5,082 (39%)
Previous	16,474 (34%)	6,143 (47%)
Current	10,256 (21%)	1,849 (14%)
Alcohol consumption (high)	11,824 (26%)	2,722 (21%)
**Medication use**		
Antiplatelet	47 (0.1%)	56 (0.4%)
Anti-hypertensive	0 (0.0%)	7,371 (56%)
Anti-coagulant	175 (0.4%)	328 (3%)
Acetylsalicylic acid	549 (1%)	1,160 (9%)
Beta-blocker	0 (0.0%)	5,378 (41%)
Calcium antagonists	0 (0.0%)	1,983(15%)
Diuretics	0 (0.0%)	4,689 (36%)
Statins	1886 (4%)	2,814 (21%)
Alternative cholesterol lowering medication	95 (0.2%)	119 (0.9%)
Anti-diabetics	592 (1%)	1,004 (8%)
**Health characteristics**		
Diagnosed hypertension	0 (0.0%)	12,291 (93%)
Diagnosed hypercholesterolemia	7,657 (16%)	3,974 (30%)
Diagnosed diabetes	1,142 (2%)	1,428 (11%)
Systolic blood pressure	136.00 [131.00, 143.00]	135.00 [125.00, 146.00]
Diastolic blood pressure	81.00 [75.00, 86.00]	78.00 [72.00, 85.00]
Total cholesterol	5.20 [4.60, 5.90]	5.20 [4.50, 5.90]
LDL cholesterol	3.44 (0.92)	3.36 (0.93)
HDL cholesterol	1.40 [1.20, 1.70]	1.40 [1.10, 1.60]
Triglycerides	1.11 [0.80, 1.58]	1.26 [0.91, 1.76]
Renal function	96.41 [86.10, 100.00]	88.45 [76.94, 97.76]

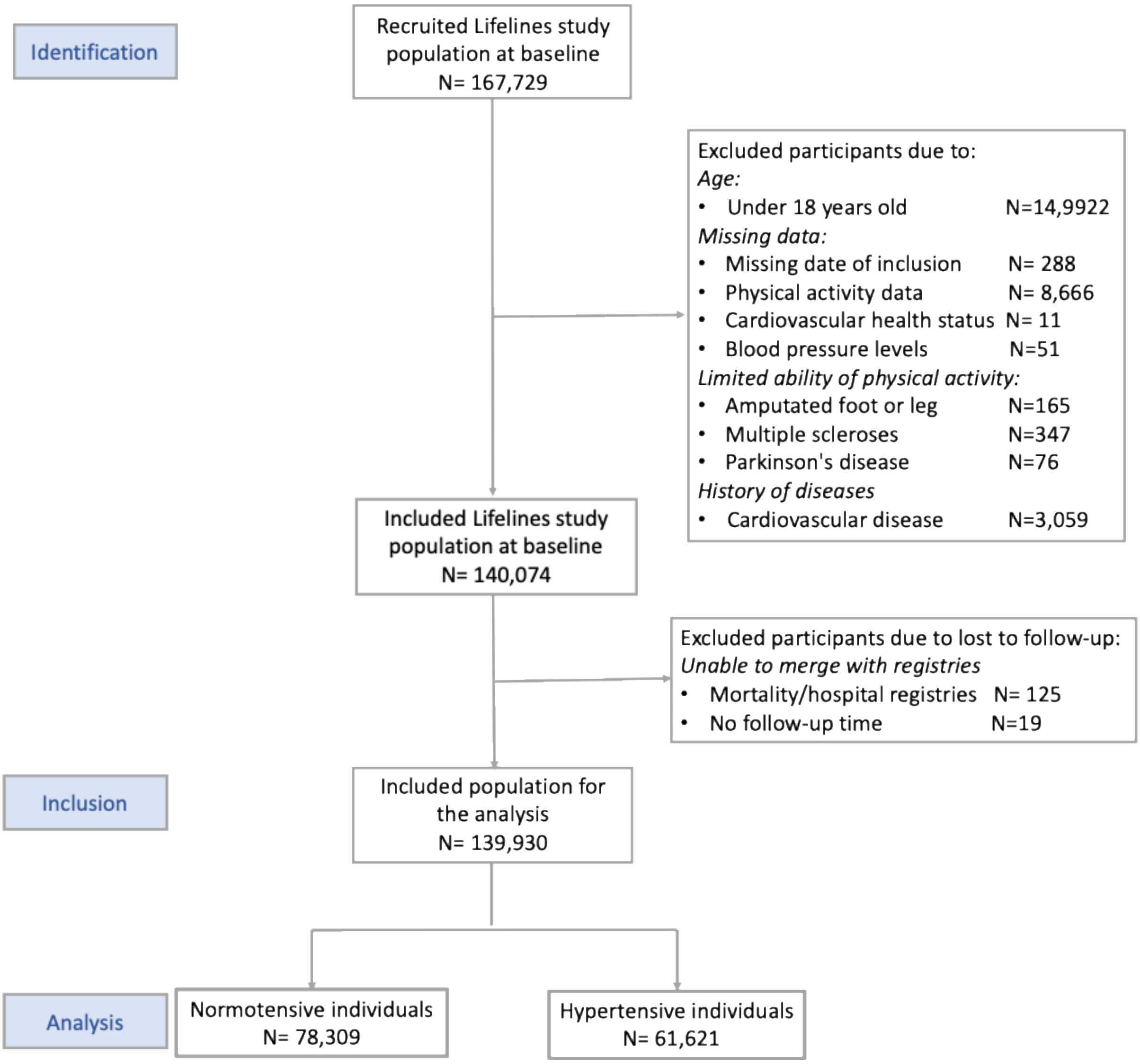

Figure S1. STROBE flow chart of included study population.
